# CD40L reverse signaling suppresses prevertebral sympathetic axon growth and tissue innervation

**DOI:** 10.1002/dneu.22735

**Published:** 2020-02-29

**Authors:** Osman Yipkin Calhan, Sean Wyatt, Alun Millward Davies

**Affiliations:** ^1^ School of Biosciences Cardiff University Cardiff UK

**Keywords:** CD40L, development, innervation, paravertebral sympathetic neuron, prevertebral sympathetic neuron, reverse signaling

## Abstract

CD40‐activated CD40L reverse signaling is a major physiological regulator of the growth of neural processes in the developing nervous system. Previous work on superior cervical ganglion (SCG) neurons of the paravertebral sympathetic chain has shown that CD40L reverse signaling enhances NGF‐promoted axon growth and tissue innervation. Here we show that CD40L reverse signaling has the opposite function in prevertebral ganglion (PVG) sympathetic neurons. During a circumscribed perinatal window of development, PVG neurons cultured from *Cd40* ^–/–^ mice had substantially larger, more exuberant axon arbors in the presence of NGF than PVG neurons cultured from wild‐type mice. Tissues that receive their sympathetic innervation from PVG neurons were markedly hyperinnervated in *Cd40* ^–/–^ mice compared with wild‐type mice. The exuberant axonal growth phenotype of cultured CD40‐deficient perinatal PVG neurons was pared back to wild‐type levels by activating CD40L reverse signaling with a CD40‐Fc chimeric protein, but not by activating CD40 forward signaling with CD40L. The co‐expression of CD40 and CD40L in PVG neurons suggests that these proteins engage in an autocrine signaling loop in these neurons. Our work shows that CD40L reverse signaling is a physiological regulator of NGF‐promoted sympathetic axon growth and tissue innervation with opposite effects in paravertebral and prevertebral neurons.

## 1 INTRODUCTION

The developing sympathetic nervous system is a well‐established model for understanding how neuron survival and axon growth are regulated and how tissue innervation is established and modified. Several extracellular signals control the establishment of sympathetic innervation in the developing peripheral nervous system by regulating neuronal survival and controlling the growth and ramification of axons within innervated tissues (Davies, 2009; Glebova & Ginty, 2005). The best characterized of these is the secreted protein nerve growth factor (NGF), which is synthesized in target tissues. NGF promotes the survival of developing sympathetic neurons and the level of NGF synthesis in different tissues regulates the number of neurons that innervate these tissues (Crowley et al., 1994; Davies, 2003). NGF also promotes the growth and branching of sympathetic axons within their target tissues (Glebova & Ginty, 2004).

Several members of the tumor necrosis factor superfamily (TNFSF) have recently been shown to be key physiological regulators of NGF‐promoted sympathetic axon growth and tissue innervation in development without affecting neuronal survival. Members of the TNFSF bind to one or more members of the TNF receptor superfamily (TNFRSF) and are active both as membrane‐integrated ligands and as soluble ligands following cleavage from the cell membrane (Grivenniko, Kuprash, Liu, & Nedospasov, 2006; Hehlgans & Pfeffer, 2005). In addition, several TNFRSF members can also act as ligands for the membrane‐integrated TNFSF to which they bind, which function as reverse signaling receptors (Sun & Fink, 2007). While the TNF and TNFR superfamilies are best understood for their many roles in the immune system (Hehlgans & Pfeffer, 2005; Sedy, Bekiaris, & Ware, 2014), several members of these superfamilies modulate NGF‐promoted sympathetic axon growth by forward and reverse signaling mechanisms. NGF‐promoted sympathetic axon growth and tissue innervation are suppressed by TNF‐activated TNFR1‐mediated forward signaling (Erice, Calhan, Kisiswa, Wyatt, & Davies, 2019; Gutierrez et al., 2013) and by BCMA‐activated TWE‐PRIL‐mediated reverse signaling (Howard et al., 2018). NGF‐promoted sympathetic axon growth and tissue innervation are enhanced by TNFR1‐activated TNF‐mediated reverse signaling (Kisiswa et al., 2013, 2017) and by CD40‐activated CD40L‐mediated reverse signaling (McWilliams, Howard, Wyatt, & Davies, 2015). GITRL/GITR signaling also enhances NGF‐promoted axon growth and tissue innervation (O'Keeffe, Gutierrez, Pandolfi, Riccardi, & Davies, 2008), although the direction of signaling, forward or reverse, has yet to be investigated.

CD40–CD40L bidirectional signaling, the focus of the current study, is of increasing interest. Not only does it play a physiological role in regulating sympathetic and sensory axon growth in the developing PNS (Howard, McWilliams, Wyatt, & Davies, 2019; McWilliams et al., 2015), it plays a key role in regulating axon and dendrite growth from several different kinds of neurons in the developing CNS (Carriba & Davies, 2017). In the immune system, CD40–CD40L signaling plays several key roles in regulating immune responses, CD40–CD40L signaling has been implicated in the pathogenesis of autoimmune disorders (Peters, Stunz, & Bishop, 2009) and there is increasing interest in the relevance of CD40–CD40L signaling for cancer immunotherapy (Richards, Sefrin, Gieffers, Hill, & Merz, 2019). This emphasizes the need to understand the biology of CD40–CD40L signaling in greater detail.

Virtually, all work on sympathetic axon growth and tissue innervation has been done on the easily accessible neurons of the superior cervical ganglion (SCG) of the paravertebral sympathetic chain and their cranial innervation targets. Here, we have studied the sympathetic neurons of prevertebral ganglia (PVG) and the visceral tissues they innervate. We find that CD40‐activated CD40L‐mediated reverse signaling is a physiologically important regulator of PVG axon growth and PVG target innervation, but with diametrically opposite functions to those observed on SCG axons and SCG target innervation. Our findings demonstrate how CD40L reverse signaling displays distinctive functions in the development of different populations of the same class of neurons.

## 2 RESULTS

### 2.1 PVG and SCG neurons co‐express CD40 and CD40L

**Figure 1:**
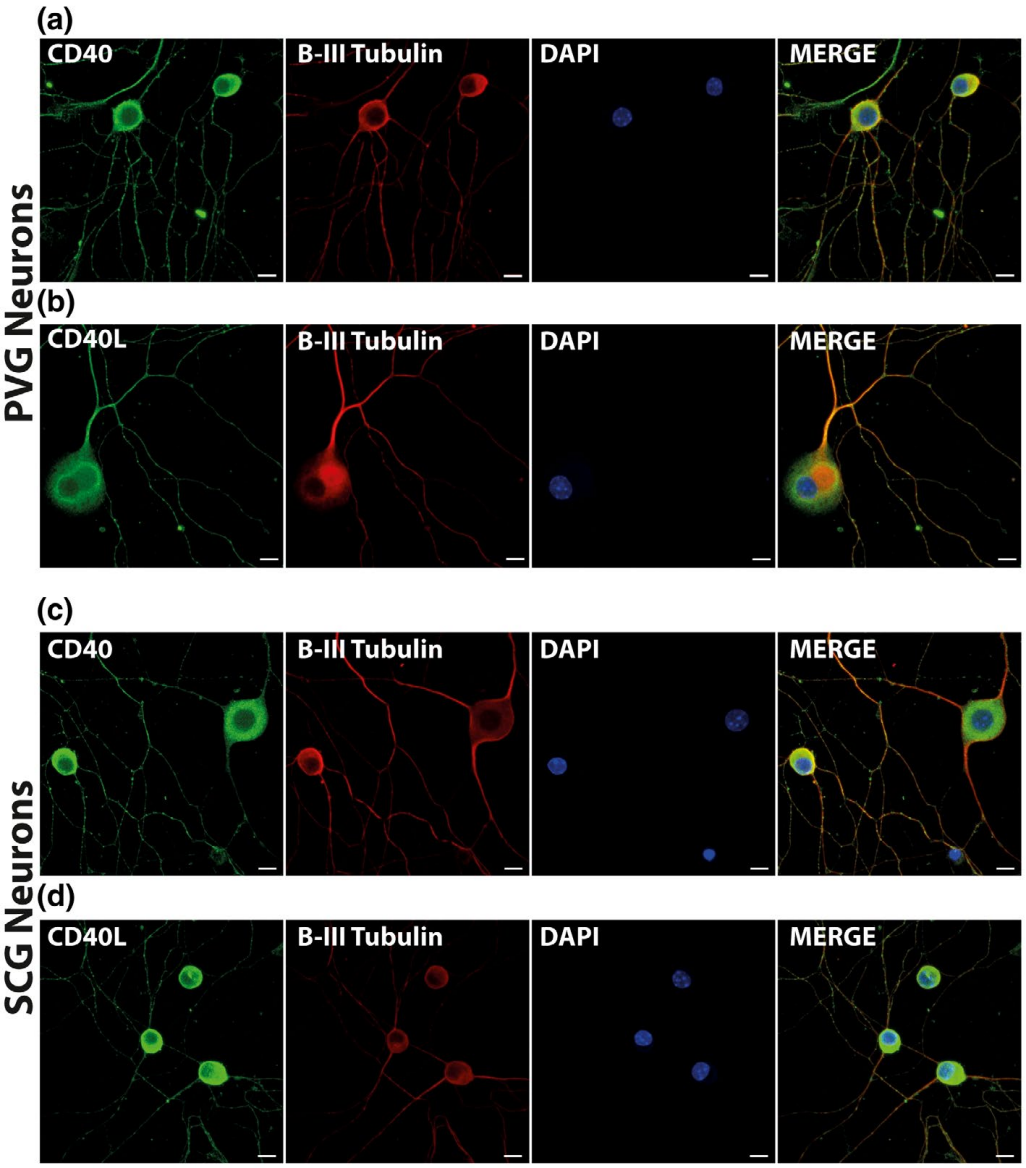
PVG and SCG neurons co‐express CD40 and CD40L. Representative images of P0 PVG neurons (a,b) and SCG neurons (c,d) cultured for 24 hr in medium containing 1 ng/ml of NGF and double labeled for either anti‐CD40 and anti‐βIII tubulin (a,c) or anti‐CD40L and anti‐βIII tubulin (b,d). The preparations were also counterstained with the nuclear marker DAPI. Scale bar, 10 μm [Color figure can be viewed at https://www.wileyonlinelibrary.com]

We have previously reported that the majority of developing SCG neurons co‐express CD40 and CD40L (McWilliams et al., 2015). We used immunocytochemistry to determine whether PVG neurons also express these glycoproteins and whether they are co‐expressed. Anti‐CD40 (Figure 1a) and anti‐CD40L (Figure 1b) antibodies each labeled the great majority of neurons positively identified by double‐labeling with anti‐βIII tubulin in dissociated cultures of P0 PVG neurons of the coeliac and superior mesenteric ganglia (90.32 ± 1.46% tubulin‐positive neurons were CD40‐positive and 92.34 ± 1.12% tubulin‐positive neurons were CD40L‐positive). For comparison, P0 SCG neuron cultures were set up in parallel and were labeled with anti‐CD40 (Figure 1c) and anti‐CD40L (Figure 1d) antibodies. Very similar labeling was observed for both antibodies (89.18 ± 2.91% tubulin‐positive neurons were CD40‐positive and 85.84 ± 2.40% tubulin‐positive neurons were CD40L‐positive), as previously reported (McWilliams et al., 2015). All of the neurons in both sets of cultures were also stained with anti‐tyrosine hydroxylase (TH), demonstrating that these were sympathetic neurons, as expected (not shown). No cells were labeled when primary antibodies were omitted and labeling with anti‐CD40 was eliminated in cultures established from *Cd40* ^–/–^ mice (not shown). These observations suggest that the great majority of PVG neurons co‐express CD40 and CD40L at the stage when sympathetic axons are innervating their targets during the peak of responsiveness to CD40L reverse signaling (see below).

### 2.2 CD40–CD40L signaling has opposing effects on NGF‐promoted axon growth from PVG and SCG neurons

The co‐expression of CD40 and CD40L by PVG neurons raised the possibility of autocrine signaling. We have previously shown that CD40–CD40L autocrine signaling in SCG neurons enhances NGF‐promoted axon growth (McWilliams et al., 2015). To ascertain how CD40L autocrine signaling might influence PVG neuron development, we compared axon growth from cultures of dissociated PVG neurons established from *Cd40* ^+/+^ and *Cd40* ^–/–^ newborn mice. We reasoned that the absence of CD40 would eliminate any potential CD40–CD40L signaling loop and reveal its role.

**Figure 2:**
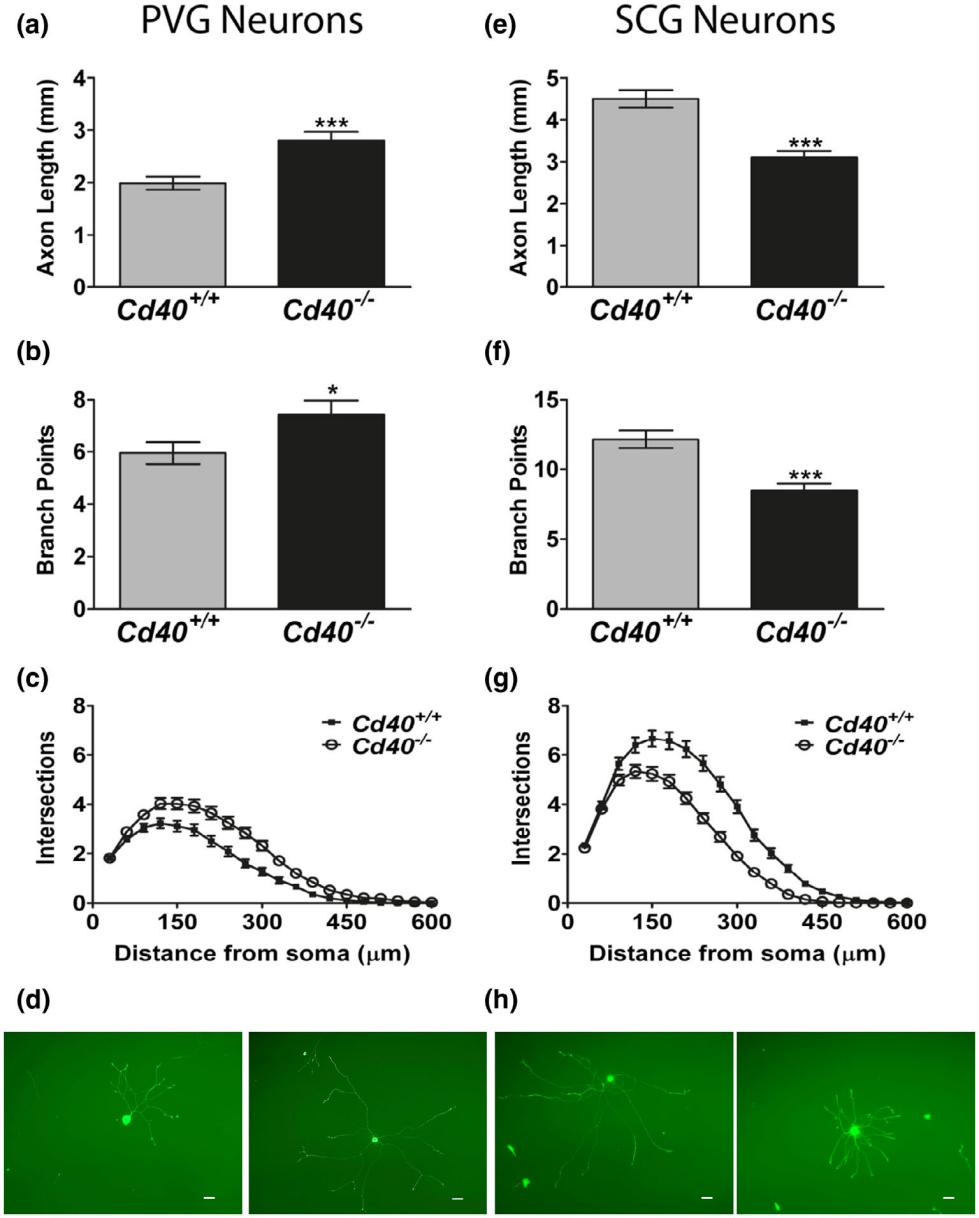
CD40–CD40L signaling has opposing effects on NGF‐promoted axon growth from PVG and SCG neurons. Total axon length (a,e), branch point number (b,f), Sholl plots (c,g) and representative images (d,h) of the neurite arbors of PVG (a–d), and SCG (e–h) neurons of P0 *Cd40* ^+/+^ and *Cd40* ^–/–^ littermates grown for 24 hr with 1 ng/ml of NGF. Mean ± *SEM* data of >150 neurons per condition combined from three experiments of each type. Scale bar, 50 μm. ***p < .001, *p < .05 (the differences between both length and branch points of *Cd40^+/+^* and *Cd40* ^–/–^ PVG and SCG neurons were analyzed by Mann–Whitney test) [Color figure can be viewed at https://www.wileyonlinelibrary.com]

To our surprise, in the presence of NGF, PVG neurons cultured from *Cd40* ^–/–^ newborn mice exhibited significantly longer and significantly more branched axon arbors than PVG neurons cultured from *Cd40* ^+/+^ littermates (Figure 2a,b). The Sholl plots for these cultures are shown in Figure 2c and representative images are shown in Figure 2d.

**Figure 3:**
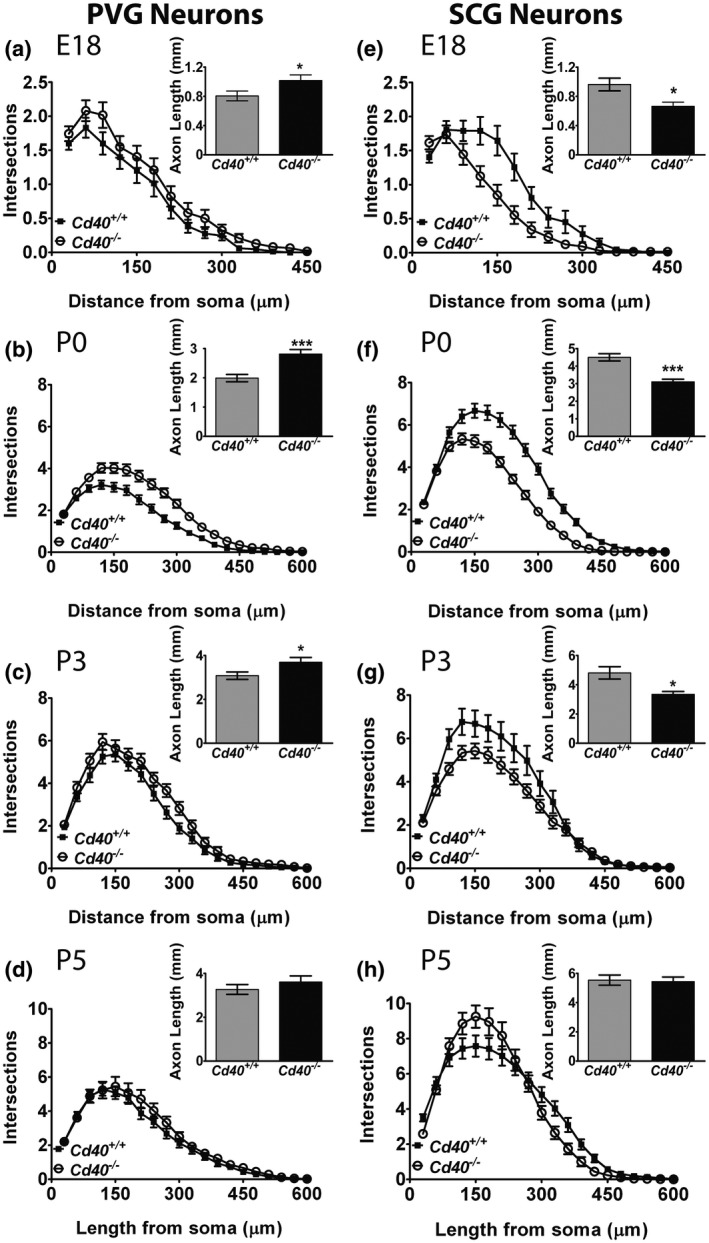
CD40–CD40L signaling modulates NGF‐promoted axon growth over the same developmental window in PVG and SCG neurons. Sholl plots PVG (a–d) and SCG (e–h) neurons at E18 (a,e), P0 (b,f), P3 (c,g) and P5 (d,h) *Cd40* ^+/+^ and *Cd40* ^–/–^ littermates grown for 24 hr with 1 ng/ml of NGF. The insets show bar charts of axon length for each age. Mean ± *SEM* data of >150 neurons per condition combined from three experiments of each type. ***p < .001, *p < .05 (the differences between both length and branch points of *Cd40^+/+^* and *Cd40* ^–/–^ PVG and SCG neurons were analyzed by Mann–Whitney test)

**Figure 4:**
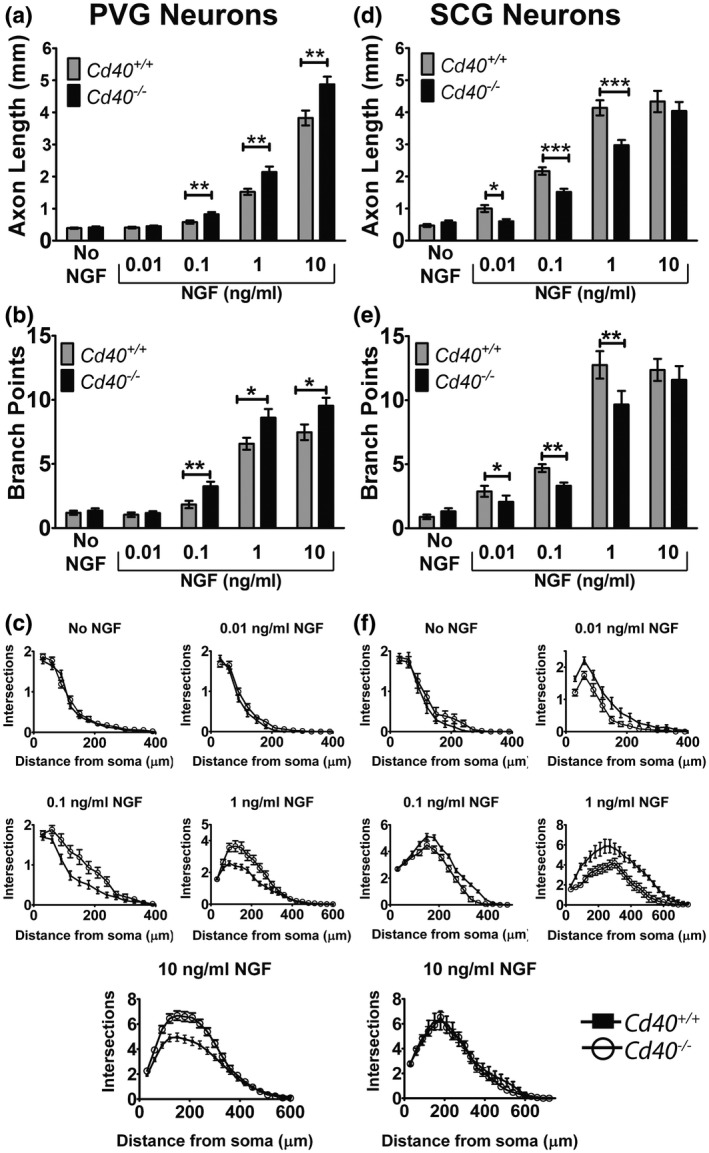
NGF concentration dependence of axon growth modulation by CD40–CD40L signaling in PVG and SCG neurons. Bar charts of total axon length (a,d) and branch point number (b,e) and Sholl plots (c,f) of the neurite arbors of PVG (a–c) and SCG (d–f) neurons of P0 *Cd40* ^+/+^ and *Cd40* ^–/–^ littermates grown for 24 hr with either no NGF or the different concentrations of NGF indicated. All cultures received 25 mM Boc‐D‐FMK to prevent apoptosis with no or low concentrations of NGF. Mean ± *SEM* data of >150 neurons per condition combined from three experiments of each type. ***p < .001, **p < .01, *p < .05. The differences between both length and branch points of *Cd40^+/+^* and *Cd40* ^–/–^ PVG and SCG neurons at each of NGF concentration points were analyzed by the Mann–Whitney test

Because this difference in axon growth from CD40‐deficient and wild‐type PVG neuron was the very opposite of what we had reported for SCG neurons cultured from *Cd40* ^+/+^ and *Cd40* ^–/–^ mice (McWilliams et al., 2015), we established dissociated cultures of PVG and SCG neurons in parallel. The SCG neuron cultures replicated our reported findings and showed that CD40‐deficient SCG neurons had shorter, less exuberant axon arbors than wild‐type SCG neurons (Figure 2e–h). These detailed comparative studies were undertaken on P0 neurons using 1 ng/ml of NGF because these are the age and NGF concentration at which the effects of CD40/CD40L signaling on axon growth are maximal in both the populations of sympathetic neurons (Figures 3 and 4). Taken together, these results suggest that CD40–CD40L signaling has opposite effects on NGF‐promoted axon growth from neonatal PVG and SCG neurons.

### 2.3 CD40–CD40L signaling modulates NGF‐promoted axon growth over the same developmental window in PVG and SCG neurons

We have reported that the effect of CD40–CD40L signaling on NGF‐promoted axon growth from SCG neurons is restricted to a developmental window and is maximal at P0 several days after the neurons have become dependent on NGF for survival when SCG axons are ramifying extensively in their targets (McWilliams et al., 2015). To explore the possibility that the effect of CD40–CD40L signaling on NGF‐promoted axon growth is also restricted to a developmental window in PVG neurons, we established dissociated PVG cultures from *Cd40* ^–/–^ mice and *Cd40* ^+/+^ littermates over a range of different ages. The difference in NGF‐promoted axon growth between PVG neurons of *Cd40* ^–/–^ mice and PVG neurons of *Cd40* ^+/+^ littermates was also observed during a clear developmental window. For direct comparison with SCG neurons, we set up cultures of PVG neurons and SCG neurons at the same ages (E18, P0, P3, and P5). For both kinds of neurons, the differences in axon length between CD40‐deficient and wild‐type neurons were maximal in cultures established at P0 (Figure 3). Smaller, statistically significant differences in axon length between CD40‐deficient and wild‐type neurons were observed in both PVG and SCG neuron cultures established at E18 (Figure 3a,e) and at P3 (Figure 3c,g). There were no statistically significant differences in axon length between CD40‐deficient and wild‐type neurons in both P5 PVG and P5 SCG cultures (Figure 3d,h). These data suggest that the regulation of NGF‐promoted axon growth by CD40–CD40L signaling operates over very similar perinatal developmental windows in both PVG and SCG neurons.

### 2.4 NGF concentration dependence of axon growth modulation by CD40–CD40L signaling in PVG and SCG neurons

Another key feature of the effect of CD40–CD40L signaling on NGF‐promoted axon growth from SCG neurons is that it was only observed over a narrow NGF concentration range between 0.01 and 1 ng/ml of NGF (McWilliams et al., 2015). To ascertain whether the effect of CD40–CD40L signaling on NGF‐promoted axon growth from PVG neurons is also NGF concentration‐dependent, we established cultures of PVG and SCG neurons in parallel from P0 *Cd40* ^–/–^ mice and *Cd40* ^+/+^ littermates and incubated them with different NGF concentrations. All cultures received the broad‐spectrum caspase inhibitor Boc‐D‐FMK to prevent apoptosis in the absence of NGF or with low levels of NGF. The great majority of neurons survived in these experiments and there were no significant differences in survival between the experimental groups (not shown).

Differences in axon length and branching between PVG neurons of *Cd40* ^–/–^ and *Cd40* ^+/+^ mice were NGF concentration‐dependent. No statistically significant differences in axon length and branching were observed in the absence of NGF and with the lowest concentration of NGF used (0.01 ng/ml). Statistically significant differences in axon length (Figure 4a) and branching (Figure 4b) between CD40‐deficient and wild‐type PVG neurons were evident in the presence of higher NGF concentrations (0.1, 1, and 10 ng/ml, the highest NGF concentration used). The NGF concentration‐dependence of axonal growth from CD40‐deficient and wild‐type SCG neurons previously reported (McWilliams et al., 2015) was repeated here. However, statistically significant differences were observed over a range of lower NGF concentrations compared with PVG neurons. No statistically significant differences in axon length and branching were observed in the absence of NGF and at the highest concentration used (10 ng/ml). Statistically significant differences in axon length (Figure 4d) and branching (Figure 4e) between CD40‐deficient and wild‐type SCG neurons were evident in cultures supplemented with a range of lower NGF concentrations (0.01, 0.1, and 1 ng/ml). The NGF concentration‐dependency of axon growth from CD40‐deficient and wild‐type PVG and SCG neurons is further illustrated by the Sholl plots for these experiments (Figure 4c,f). Taken together, these results suggest that axon growth differences between CD40‐deficient and wild‐type neurons are NGF concentration‐dependent for both PVG and SCG neurons, but the NGF concentration was shifted upward by an order of magnitude for PVG neurons.

### 2.5 Regulation of *Cd40* mRNA expression in PVG and SCG neurons by NGF

**Figure 5:**
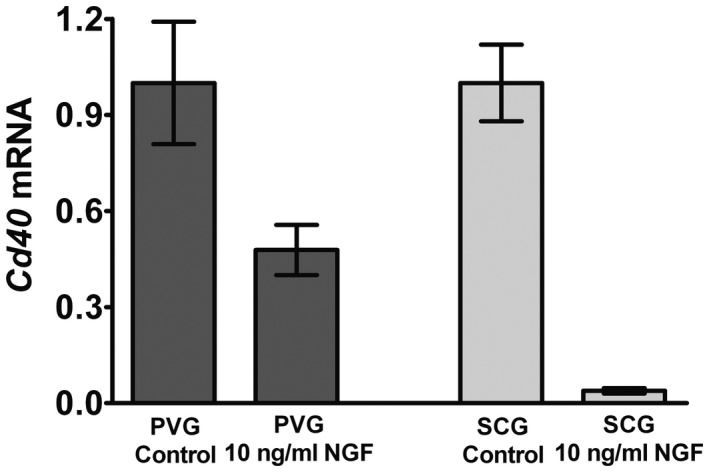
Regulation of *Cd40* mRNA expression in PVG and SCG neurons by NGF. Bar chart of the levels of *Cd40* mRNA relative to reference mRNAs in wild‐type cultures of P0 PVG and SCG neurons cultured for 24 hr with and without 10 ng/ml of NGF. The data are normalized to a value of 1.0 for PVG neurons and for SCG neurons cultured without NGF. All cultures received 25 mM Boc‐D‐FMK. Mean ± *SEM* of data from three separate experiments

The lack of effect of *Cd40* deletion on axon growth from SCG neurons at a high NGF concentration has been shown to be due to a marked negative regulation of *Cd40* mRNA expression by NGF in these neurons (McWilliams et al., 2015). To ascertain whether NGF regulates the expression of *Cd40* mRNA in PVG neurons and to compare with the effect of NGF on the expression of *Cd40* mRNA in SCG neurons, we cultured both kinds of neurons at P0 with and without 10 ng/ml of NGF. To ensure similar survival in both culture conditions, all cultures received Boc‐D‐FMK. The measurement of *Cd40* mRNA by RT‐qPCR after 24 hr showed that NGF caused a very marked 25‐fold reduction of *Cd40* mRNA in SCG cultures but only a 2‐fold reduction in PVG cultures (Figure 5). This suggests that NGF negatively regulates the expression of *Cd40* mRNA in both kinds of neurons, but that SCG neurons are substantially more sensitive to negative regulation by NGF than PVG neurons.

### 2.6 Modulation of NGF‐promoted axon growth from PVG and SCG neurons by a CD40L reverse signaling mechanism

**Figure 6:**
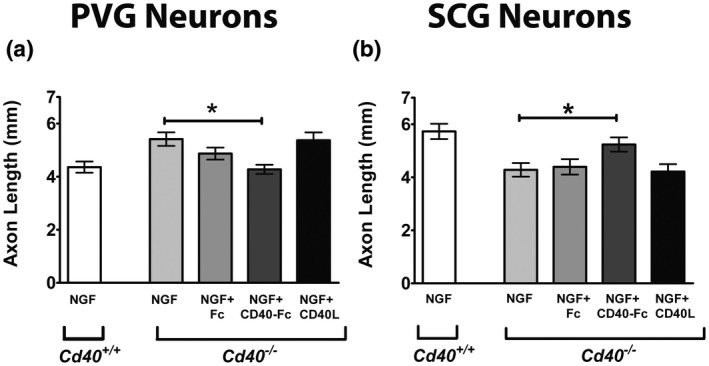
Modulation of NGF‐promoted axon growth from PVG and SCG neurons by a CD40L reverse signaling mechanism. Bar charts of total axon lengths in the arbors of PVG (a) and SCG (b) neurons of P0 *Cd40* ^+/+^ and *Cd40* ^–/–^ littermates grown for 24 hr with either 1 ng/ml of NGF alone or NGF plus either 1 μg/ml of Fc, 1 μg/ml of CD40‐Fc or 1 μg/ml of CD40L. Mean ± *SEM* data of >150 neurons per condition combined from three experiments of each type. *p < .05, one way ANOVA Kruskal–Wallis with Dunn's post hoc

Interaction between CD40L and CD40 can initiate bidirectional signaling: CD40L‐activated CD40‐mediated forward signaling and CD40‐activated CD40L‐mediated reverse signaling (Sun & Fink, 2007). In vitro phenotype rescue experiments have demonstrated that CD40L reverse signaling mediates enhanced NGF‐promoted axon growth from SCG neurons (McWilliams et al., 2015). To ascertain whether either CD40 forward signaling or CD40L reverse signaling mediates suppression of NGF‐promoted axon growth from PVG neurons, we established NGF‐supplemented PVG neuron cultures from P0 *Cd40* ^+/+^ and *Cd40* ^–/–^ littermates. The aim was to ascertain whether CD40L or CD40‐Fc chimera (in which the extracellular domain of CD40 is linked to the Fc part of human IgG1) that activates CD40L‐mediated reverse signaling (Carriba & Davies, 2017; McWilliams et al., 2015) could rescue the exuberant axon phenotype of CD40‐deficient PVG neurons. As a control, NGF‐supplemented SCG cultures from P0 *Cd40* ^+/+^ and *Cd40* ^–/–^ littermates were set up in parallel and were treated with CD40L and CD40‐Fc. Compared with wild‐type neurons, the axon arbors of CD40‐deficient PVG and CD40‐deficient SCG neurons were significantly larger and smaller, respectively, than the axon arbors of wild‐type neurons (Figure 6a,b). In both PVG and SCG neurons, these phenotypic changes were pared back to wild‐type levels by CD40‐Fc but not by CD40L. These results suggest that the contrasting effects of CD40–CD40L signaling on NGF‐promoted axon growth from PVG and SCG neurons are mediated by reverse signaling in both kinds of neurons.

### 2.7 Hyperinnervation of PVG target organs in mice with a null mutation in the *Cd40* gene

To ascertain the physiological significance of our in vitro observations, we used immunolabeling‐enabled three‐dimensional imaging of solvent‐cleared organs (iDISCO) (Renier et al., 2014) to visualize and compare the innervation of selected organs in *Cd40* ^–/–^, *Cd40* ^±^, and *Cd40* ^+/+^ littermates that were generated by crossing *Cd40* ^±^ mice. Axons were labeled using an antibody against tyrosine hydroxylase (TH), a rate‐limiting enzyme in the biosynthesis of noradrenaline, a marker for sympathetic fibers. We examined three organs that receive their sympathetic innervation predominantly from PVG neurons in rodents, the stomach, spleen, and kidney. The stomach receives its sympathetic innervation predominantly from the coeliac and superior mesenteric ganglia and has smaller contributions from the splanchnic ganglia and the lower thoracic paravertebral sympathetic chain. The spleen receives its sympathetic innervation predominantly from the splanchnic ganglia and coeliac ganglion and has smaller contributions from the inferior mesenteric ganglion and lower thoracic paravertebral sympathetic chain. Most of the sympathetic neurons that innervate the kidney are located in the coeliac ganglion (Chevendra & Weaver, 1991; Gattone, Marfurt, & Dallie, 1986; Quinson, Robbins, Clark, & Furness, 2001; Trudrung, Furness, Pompolo, & Messenger, 1994).

Although the great majority of TH‐positive fibers in abdominal organs originate from sympathetic ganglia, the nodose ganglion, which contributes to the sensory innervation of abdominal and thoracic viscera, does contain a small subset of TH‐positive neurons in rodents (Katz, Markey, Goldstein, & Black, 1983; Zhuo, Ichikawa, & Helke, 1997) and some TH‐positive nodose fibers have been convincingly demonstrated in at least the stomach (Kummer, Bachmann, Neuhuber, Hanze, & Lang, 1993). Because of this, any effect of CD40/CD40L signaling on axon growth and branching from nodose neurons could potentially interfere to some extent with our in vivo analysis. Notwithstanding the small number of TH‐positive nodose fibers involved, we carried out experiments on dissociated nodose ganglion neurons cultured from *Cd40* ^+/+^ and *Cd40* ^–/–^ mice. Because the great majority of mouse nodose ganglion neurons depend on BDNF for survival and because nodose neurons have an earlier developmental profile than sympathetic neurons (Davies, Lee, & Jaenisch, 1993), the responses of late fetal nodose neurons to a broad range of BDNF concentrations (0.01 to 10 ng/ml) were analyzed. However, the BDNF dose responses of *Cd40* ^+/+^ and *Cd40* ^–/–^ nodose neurons were overlapping and virtually identical (not shown), suggesting that target innervation by TH‐positive nodose ganglion neurons is unlikely to affect our analysis.

**Figure 7:**
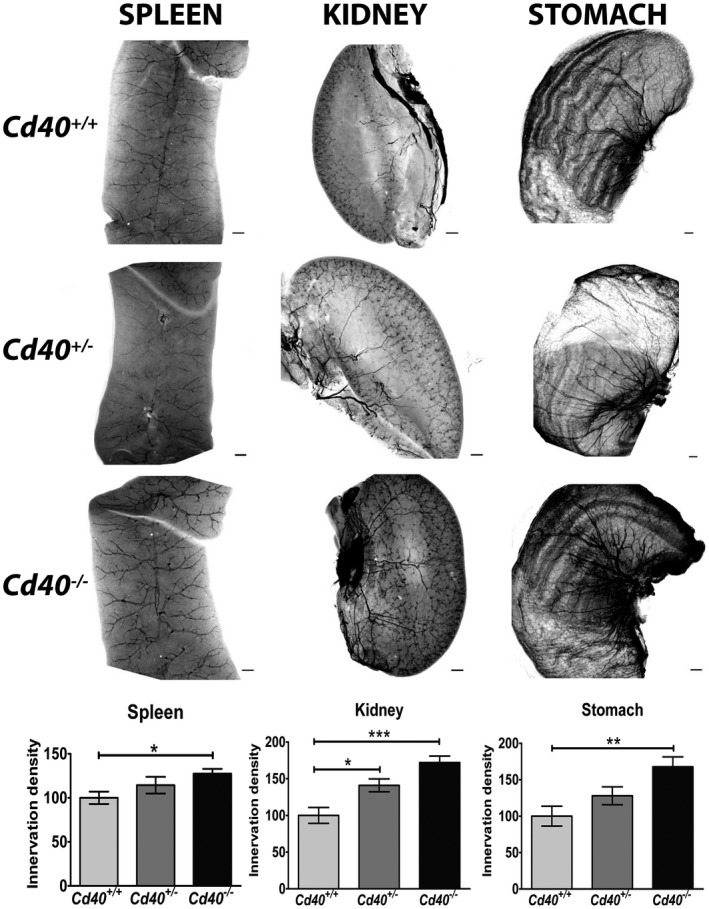
Hyperinnervation of PVG target organs in mice with a null mutation in the *Cd40* gene. Representative images of iDISCO preparations of the spleen (a), kidney (b) and stomach (c) of P10 *Cd40* ^+/+^, *Cd40* ^±^, and *Cd40* ^–/–^ littermates stained for TH. Scale bars, 100 μm. Bar charts of innervation density quantified in the spleen (d), kidney (e) and, stomach (f) in at least six mice of each genotype normalized to 100 in *Cd40* ^+/+^ mice. ***p < .001, **p < .01, and *p < .05, one way ANOVA with Dunnett's post hoc

The mice were studied using iDISCO at P10, by which time the sympathetic innervation of these organs has become well established. The representative images shown in Figure 7a–c show that the spleen, kidney, and stomach were each more densely innervated in *Cd40* ^–/–^ mice compared with *Cd40* ^+/+^ mice. Quantification of a standardized region of each organ performed blind showed that the innervation density of each organ was significantly higher in *Cd40* ^–/–^ mice compared with *Cd40* ^+/+^ littermates (Figure 7d–f). In all organs, the innervation density of these organs in *Cd40* ^±^ mice was intermediate between that of *Cd40* ^–/–^ and *Cd40* ^+/+^ littermates; however, only in the kidney was innervation density of *Cd40* ^±^ mice significantly greater than that of *Cd40* ^+/+^ littermates. These in vivo observations suggest that CD40 is a physiologically relevant regulator of PVG target innervation. These observations are consistent with our in vitro data and suggest that CD40 functions as a negative regulator of PVG target innervation.

### 2.8 NGF mRNA levels in PVG and SCG target tissues

**Figure 8:**
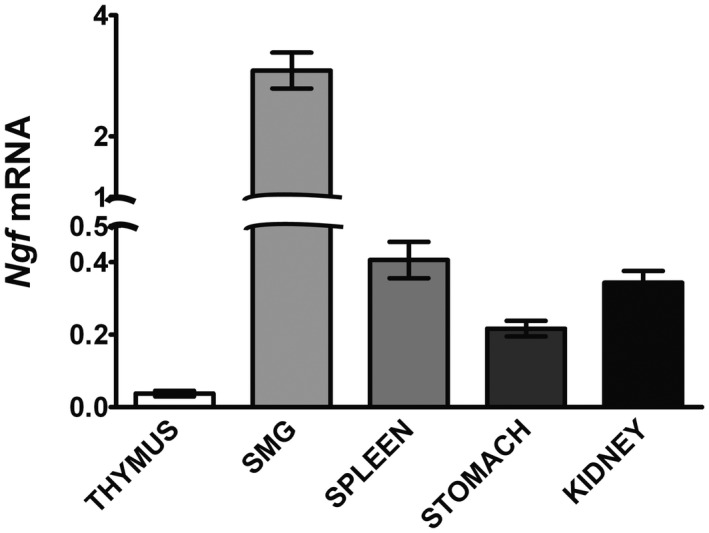
NGF mRNA levels in PVG and SCG targets. Bar chart of the levels of *Ngf* mRNA relative to reference mRNAs in the thymus, submandibular salivary gland (SMG), spleen, stomach, and kidney dissected from P10 wild‐type mice

We have shown that *Cd40* ^–/–^ mice have reduced sympathetic innervation in only a subset of SCG targets: those that express a very low level of NGF, whereas those that have a high level of NGF are unaffected (McWilliams et al., 2015). To assess the relative level of NGF synthesis in PVG targets and compare this with different SCG targets we measured the relative levels of NGF mRNA in different tissues at P10 relative to reference mRNAs encoding housekeeping proteins (glyceraldehyde phosphate dehydrogenase, succinate dehydrogenase, and hypoxanthine phosphoribosyltransferase‐1). In addition to PVG targets (spleen, stomach, and kidney), we measured the level of NGF mRNA in two SCG targets: the thymus which expresses a very low level of NGF and whose innervation is significantly reduced in *Cd40* ^–/–^ mice and the submandibular salivary gland (SMG) which expresses a very high level of NGF and whose innervation is unaffected in *Cd40* ^–/–^ mice (McWilliams et al., 2015). Figure 8 shows that the level of NGF mRNA was substantially higher in the SMG than in the thymus (about 130‐fold higher) whereas the levels of NGF mRNA in the spleen, stomach, and kidney were much higher than in the thymus (between 5‐ and 10‐fold greater) but much lower than in the SMG. Our findings suggest that the NGF mRNA level in PVG targets is intermediate between tissues whose innervation by the SCG is either reduced or unaffected by the deletion of the *Cd40* gene.

## 3 DISCUSSION

Our analysis of mice with a null mutation in the *Cd40* gene has demonstrated that CD40 is a physiological regulator of the establishment of the sympathetic innervation of organs innervated by PVG. We show that all PVG target organs examined in *Cd40* ^–/–^ mice are hyperinnervated compared with those in wild‐type mice. This is in marked contrast to the subset of SCG targets that have been shown to be hypoinnervated in *Cd40* ^–/–^ mice (McWilliams et al., 2015). This difference in the sympathetic innervation of PVG and SCG targets in CD40‐deficient mice is consistent with our in vitro studies, which demonstrate that CD40 has opposite effects on NGF‐promoted axon growth from PVG and SCG neurons. Whereas the axon arbors of PVG neurons cultured from CD40‐deficient mice are significantly longer and more branched than wild‐type PVG neurons, the axon arbors of SCG neurons cultured from CD40‐deficient neonates are significantly shorter and less branched than wild‐type SCG neurons. This suggests that CD40 reduces NGF‐promoted axon growth from PVG neurons, whereas CD40 enhances NGF‐promoted axon growth from SCG neurons. Interestingly, PVG neurons are slightly less responsive to NGF than SCG neurons at all ages studied. The reason for this small consistent difference is unclear. This difference does not affect our conclusion that CD40 has opposite roles in the regulation of NGF‐promoted axon growth in PVG and SCG neurons.

The co‐expression of CD40 and CD40L in both PVG and SCG neurons and our demonstration that the NGF‐promoted axon growth phenotype of both CD40‐deficient PVG and SCG neurons is rescued by CD40‐Fc, but not by CD40L, suggests that CD40 affects axon growth by participating in an autocrine CD40L reverse signaling loop in both kinds of sympathetic neurons. Several other members of the TNF and TNFR superfamilies modulate axon growth by acting via autocrine signaling loops (Gavalda, Gutierrez, & Davies, 2009; Howard et al., 2018; Kisiswa et al., 2013; O'Keeffe et al., 2008). It is only by experimentally breaking such loops, by either knocking out one of the participants of the signaling loop or by function‐blocking antibodies that interfere with binding of the participants, that the importance of these loops has been revealed.

In addition to regulating axon growth by participating in an autocrine CD40L reverse signaling loop in both PVG and SCG neurons, CD40 displays several other similarities of axon growth regulation in both of these populations of sympathetic neurons. First, it regulates axon growth during the same brief perinatal window of development in PVG and SCG neurons, a stage after the neurons have become dependent on NGF for survival and when their axons are ramifying extensively within their targets. Other TNF/TNFR superfamily members that regulate axon growth usually act over similar late developmental windows (Gavalda et al., 2009; Gutierrez et al., 2013; Howard et al., 2018; Kisiswa et al., 2013; O'Keeffe et al., 2008). Second, CD40L reverse signaling does not affect axon growth on its own in either PVG or SCG neurons, rather it modulates the response of both kinds of neurons to the axon growth‐promoting effects of the neurotrophin NGF. This is similar to several other TNF/TNFR superfamily members that also modulate the axon growth‐promoting actions of neurotrophins, either positively or negatively, but do not affect axon growth alone (Gutierrez et al., 2013; Howard et al., 2018; O'Keeffe et al., 2008; Wheeler et al., 2014). Third, CD40L reverse signaling has no effect on the survival of PVG and SCG neurons, either alone or in the presence of NGF. CD40L reverse signaling only modulates the axon‐promoting actions of NGF, not the survival‐promoting actions of NGF. In this respect, CD40L reverse signaling in PVG and SCG neurons is similar to other TNF and TNFR superfamily members that regulate axon growth. These proteins, unlike neurotrophins, have no effect on neuronal survival but selectively modulate the axon growth‐promoting actions of neurotrophins (Gavalda et al., 2009; Gutierrez et al., 2013; Howard et al., 2018; Kisiswa et al., 2013; O'Keeffe et al., 2008).

It is striking that CD40L reverse signaling has opposite effects on NGF‐promoted axon growth from different populations of the same class of neurons. In this respect, there is a parallel with the opposite effects of CD40L reverse signaling on dendrite growth from different kinds of CNS neurons. Whereas CD40L reverse signaling is a physiological enhancer of dendrite growth and branching from hippocampal pyramidal neurons, it is a physiological repressor of dendrite growth and branching from striatal medium spiny neurons (Carriba & Davies, 2017). However, it is not known whether CD40L reverse signaling modulates the actions of a putative dendrite growth‐promoting factor or whether it modulates an intrinsic program of dendrite growth in these different CNS neurons. In future work, it will be informative to elucidate the molecular mechanisms by which CD40L reverse signaling exerts diametrically opposite effects on neural process growth from different neurons.

Another aspect that our work on CD40L reverse signaling in developing sympathetic neurons has begun to clarify is how the level of NGF itself regulates the operation of the CD40–CD40L signaling loop. We have shown that NGF negatively regulates *Cd40* mRNA expression in both PVG and SCG neurons, which, in turn, decreases the influence of the CD40–CD40L signaling loop on axon growth and target innervation density. SCG neurons are an order of magnitude more sensitive to the negative regulation of CD40 expression by NGF than PVG neurons. The in vivo consequence of this is that the innervation of SCG targets expressing high levels of NGF is not at all affected in *Cd40* ^–/–^ mice (McWilliams et al., 2015). The inference of this observation is that the growth‐promoting CD40–CD40L signaling loop is switched off in the SCG neurons that innervate these targets. Defective innervation in *Cd40* ^–/–^ mice is only observed in SCG targets that express vanishingly low levels of NGF (McWilliams et al., 2015), presumably because there is insufficient NGF to switch off the growth‐promoting CD40–CD40L signaling loop in the neurons that innervate these targets in vivo. The PVG targets we studied express levels of NGF mRNA that are intermediate between the very high and very low levels of NGF mRNA in the SCG targets we previously studied. The level of NGF synthesis in these PVG targets is insufficient to repress the CD40–CD40L signaling loop in PVG neurons because there is a hyperinnervation phenotype in these targets in *Cd40* ^–/–^ mice. Nonetheless, it is likely that the negative regulation of CD40–CD40L signaling by target‐derived NGF in PVG neurons may have subtle regional consequences for the innervation of different targets depending on the level of NGF they synthesize. In future work, it will be interesting to explore this relationship in‐depth by examining the sympathetic innervation of a variety of PVG targets that express different levels of NGF in *Cd40* ^–/–^ mice.

Because of the difficulties of culturing PVG neurons, there has only been one previous comparative study of the regulation of axon growth from prevertebral and paravertebral sympathetic neurons (Erice et al., 2019). Whereas TNFR1‐activated TNF‐mediated reverse signaling increases NGF‐promoted axon growth from SCG neurons, it does not affect PVG axon growth. Accordingly, SCG targets, but not PVG targets, are hypoinnervated in mice that lack either TNFR1 or TNF. These findings together with our current observations are beginning to reveal unsuspected physiologically relevant differences in the regulation of axon growth from prevertebral and paravertebral neurons. This raises the question of whether differences in axon growth regulation between SCG and PVG neurons apply to other growth factors. Comparative studies of the response of SCG and PVG neurons to factors that have been shown to affect SCG axon growth may provide a clearer understanding of how the sympathetic innervation of different tissues is established and refined during development.

In addition to the widespread influence of CD40L‐mediated CD40‐activated reverse signaling on axon and dendrite growth, CD40L‐activated CD40‐mediated forward signaling has recently been shown to be a physiological regulator of axon growth from developing sensory neurons (Howard et al., 2019). In contrast to the late developmental window over which CD40L reverse signaling and other members of TNF/TNFR superfamilies influence the growth of neural processes, CD40 forward signaling acts very early and transiently on sensory axons when they are growing to their targets. In addition to acting by an autocrine mechanism, there is evidence for a significant target‐derived component to the action of CD40 forward signaling on early sensory neurons (Howard et al., 2019).

Our current study adds to the growing body of work showing that CD40 and CD40L play an extraordinarily wide physiological role in regulating the growth of neural processes in the developing nervous system. CD40 and CD40L influence axon and dendrite growth from several kinds of PNS and CNS neurons at different key stages of development, acting by reverse and forward signaling mechanisms. The unique feature revealed by our current study is the surprising finding that CD40–CD40L can have marked and diametrically opposite effects on different populations of the same class of neurons and the innervation of their target tissues. While all studies of the influence of CD40L and CD40 signaling have focused on normal neural development, it will be interesting to explore the possibility that CD40L and CD40 play a role modulating the morphology of neural processes in the mature nervous system. It will be particularly interesting to explore whether CD40L‐CD40 signaling participates in regeneration following neural injury and disease, especially given the well‐established role of CD40L‐CD40 signaling in the immune system and in autoimmune disease (Calderhead, Kosaka, Manning, & Noelle, 2000; Peters et al., 2009).

## 4 MATERIALS AND METHODS

### 4.1 Mice

Mice were housed in a 12 hr light–dark cycle with access to food and water ad libitum. Breeding was approved by the Cardiff University Ethical Review Board and was performed within the guidelines of the Home Office Animals (Scientific Procedures) Act, 1986. *Cd40* null mutant mice on a C57BL6/J background were purchased from The Jackson Laboratory (Maine, USA). These mice were back‐crossed into a CD1 background. *Cd40* ^±^ mice were crossed to generate *Cd40* ^–/–^, *Cd40* ^±^, and *Cd40* ^+/+^ mice.

### 4.2 Immunocytochemistry

The cultures were fixed in 4% paraformaldehyde (PFA) for 20 min and were washed with phosphate‐buffered saline (PBS) before blocking nonspecific binding and permeabilizing the cells with 5% bovine serum albumin (BSA) and 5% donkey serum plus 0.1% Triton X‐100 (Sigma, Dorset, UK) in PBS for 1 hr at room temperature. Neurons were incubated overnight with the primary antibody in 1% blocking solution at 4°C. After washing with PBS, the cultures were incubated with the appropriate secondary antibody. The primary antibodies were: anti‐class III β tubulin (1:500, Abcam, ab41489), anti‐CD40 (1:500, MA5‐15535, Invitrogen), and anti‐CD40L (1:200, Abcam, ab2391). Secondary antibodies were Alexa Fluor conjugated anti‐immunoglobulin from Thermo Fisher Scientific used at 1:500 (donkey anti‐rabbit IgG Alexa Fluor 488, A32790, goat anti‐chicken Alexa Fluor 594, A11042 and goat anti‐mouse Alexa Fluor 488, A11001). Images were obtained using a Zeiss LSM 710 confocal microscope.

### 4.3 Dissociated neuron culture

Dissected paravertebral SCG and prevertebral coeliac and superior mesenteric ganglia (PVG) were freed of adherent connective tissue using tungsten needles and were trypsinized and plated at very low density (~200 neurons per dish/well) in poly‐ornithine and laminin‐coated 35 mm tissue culture dishes (Greiner, Gloucestershire, UK) or 4‐well dishes (Starlab, Milton Keynes, UK) in serum‐free Hams F14 medium (Davies et al., 1993) supplemented with 0.25% Albumax I (Invitrogen, Paisley, UK). Neurons were grown with NGF, Fc control protein, recombinant CD40‐Fc, CD40L (R&D Systems), and caspase inhibitor III (Boc‐D‐FMK) (Merck) at the concentrations indicated. Analysis of the size and complexity of neurite arbors was carried out in 35 mm dishes 24 hr after plating. The neurite arbors were labeled by incubating the neurons with the fluorescent vital dye calcein‐AM (1:1,000, Invitrogen, Paisley, UK) at the end of the experiment. Images of neurite arbors were acquired by fluorescence microscopy and analyzed to obtain the Sholl profiles (Gutierrez & Davies, 2007).

### 4.4 Quantification of sympathetic innervation

Several organs that receive sympathetic innervation predominantly from PVG (stomach, spleen, and kidney) were dissected and processed for the visualization of sympathetic fibers by tyrosine hydroxylase (TH) staining using the immunolabeling‐enabled three‐dimensional imaging of solvent‐cleared organs (iDISCO) technique. The experiments were carried out on tissues from P10 pups. Batches of tissue from littermates of all three genotypes were processed at the same time to ensure they were stained in an identical manner. TH‐positive fibers were fluorescently labeled using anti‐TH antibody (1:200 dilution of antibody AB152, Chemicon) and secondary antibody 1:300 (donkey anti‐rabbit IgG Alexa Fluor 594, A21207, Thermo Fisher Scientific). Using a 3‐D printed compartment, the stomach, spleen, and kidney were imaged in their entirety using confocal microscopy (Zeiss Axio Z2 Imager) and z‐stacks were constructed. The same anatomical regions from each organ set were analyzed to keep the quantification consistent. The kidney samples were each analyzed entirely. The spleen samples curled up from the anterior and posterior ends, so to exclude variability that imaging the small curled regions, these parts were excluded, keeping the analyzed surface area the same in all spleen samples. The stomach has milk at this stage of the development which causes an uneven surface near the greater curvature for standardized quantification. To provide the same measurement criteria for the analysis, we chose the most intensely innervated regions: the lesser curvature, cardia, fundus, and upper half of the body. Fiji‐Image J was used for the semi‐automated quantification of TH‐immunoreactivity as a measure of sympathetic target organ innervation density. Images were converted to greyscale and the Feature Extraction (FeatureJ Hessian) tool was employed using the smallest eigenvalue of Hessian tensor with the smoothing scale set to 1.5. To ensure consistent analysis across all conditions, multiple images from all mice were initially analyzed to generate a uniform threshold value, which was applied to every image of the same organ analyzed followed by a user‐defined macro to provide a quantitative measurement of TH‐immunoreactivity within each target organ. The data are expressed as a percentage of the mean of the wild‐type data. All imaging and quantification were performed blind.

### 4.5 RT‐qPCR

The levels of *Ngf* and *Cd40* mRNAs were quantified by real‐time PCR relative to a geometric mean of mRNAs for the housekeeping enzymes glyceraldehyde phosphate dehydrogenase (*Gapdh*), succinate dehydrogenase (*Sdha*), and hypoxanthine phosphoribosyltransferase‐1 (*Hprt1*). Total RNA was extracted from dissected sympathetic target fields or cultured sympathetic neurons using the RNeasy lipid mini extraction kit (Qiagen, Crawley, UK) and 5 μl was reverse transcribed for 1 hr at 45°C using the AffinityScript kit (Agilent, Berkshire, UK) in a 25 μl reaction according to the manufacturer's instructions. About 2 μl of cDNA was amplified in a 20 μl reaction volume using Brilliant III ultrafast qPCR master mix reagents (Agilent). PCR products were detected using dual‐labeled (FAM/BHQ1) hybridization probes specific to each of the cDNAs (MWG/Eurofins, Ebersberg, Germany). The PCR primers were: *Ngf* forward: 5ʹ‐AAA CGG AGA CTC CAC TCA CC‐3ʹ and reverse: 5ʹ‐GTC CTG TTG AAA GGG ATT GTA CC‐3ʹ; *Cd40* forward: 5ʹ‐CTT TGG AGT TAT GGA GAT G‐3ʹ and reverse: 5ʹ‐ATG ACT GAT TGG AGA AGA‐3ʹ; *Gapdh* forward: 5ʹ‐GAG AAA CCT GCC AAG TAT G‐3ʹ and reverse: 5ʹ‐GGA GTT GCT GTT GAA GTC‐3ʹ; *Sdha* forward: 5ʹ‐GGA ACA CTC CAA AAA CAG‐3ʹ and reverse: 5ʹ‐CCA CAG CAT CAA ATT CAT‐3ʹ; *Hprt1* forward: TTA AGC AGT ACA GCC CCA AAA TG and reverse: AAG TCT GGC CTG TAT CCA ACA C. Dual‐labeled probes were: *Ngf*: 5ʹ‐FAM‐TGT TCA GCA CCC AGC CTC CAC CCA‐BHQ1‐3ʹ; *Cd40*: 5ʹ‐FAM‐CCA CTG AGA CCA CTG ATA CCG‐BHQ1‐3ʹ; *Gapdh*: 5ʹ‐FAM‐AGA CAA CCT GGT CCT CAG TGT‐BHQ1‐3; *Sdha*: 5′‐FAM‐CCT GCG GCT TTC ACT TCT CT‐BHQ1‐3ʹ, *Hrpt1*: FAM‐TCG AGA GGT CCT TTT CAC CAG CAA G‐BHQ1. Forward and reverse primers were used at a concentration of 150 nM and dual‐labeled probes were used at a concentration of 300 nM. PCR was performed using the Mx3000P platform (Agilent) using the following conditions: 45 cycles of 95°C for 10 s and 60°C for 35 s. Standard curves were generated for each cDNA for every real‐time PCR run, by serial fivefold dilutions of reverse‐transcribed mouse adult spleen total RNA (Zyagen, San Diego, USA). Relative mRNA levels were quantified in four separate sets of dissected tissues and cultured cells for each experiment. Primer and probe sequences were designed using Beacon Designer software (Premier Biosoft, Palo Alto, USA).

### 4.6 Statistics

Statistical comparisons for normally distributed data were performed by *t*‐test or one‐way ANOVA followed by Dunnett's post hoc test. Non‐parametric Mann–Whitney test or one‐way ANOVA Kruskal–Wallis test followed by the Dunn's multiple post hoc test were performed for the data that were not normally distributed.

## CONFLICT OF INTEREST

The authors declare no competing or financial interests.

## AUTHOR CONTRIBUTIONS

O.Y.C. carried out the in vitro experiments and made the in vivo observations, S.W. carried out the RT‐qPCR, and A.M.D. wrote the manuscript.

## Data Availability

The data that support the findings of this study are available from the corresponding author upon reasonable request.
